# Ontogenetic Change in Behavioral Responses to Structural Enrichment From Fry to Parr in Juvenile Atlantic Salmon (*Salmo salar* L.)

**DOI:** 10.3389/fvets.2021.638888

**Published:** 2021-07-26

**Authors:** Ingeborg Bjerkvik Alnes, Knut Helge Jensen, Arne Skorping, Anne Gro Vea Salvanes

**Affiliations:** Department of Biological Sciences, University of Bergen, Bergen, Norway

**Keywords:** natal habitat enrichment, sensitive life stage, experience, exploratory behavior, stress coping behavior

## Abstract

Enrichment is widely used as a tool for studying how changes in environment affect animal behavior. Here, we report an experimental study investigating if behaviors shaped by stimuli from environmental enrichment depending on the stage animals are exposed to enrichment. We used juvenile Atlantic salmon (*Salmo salar*) in their first autumn. This is a species commonly reared for conservation purposes. Previous work has shown that environmental enrichment had no effect on long-term survival when the fry stage (smaller than 70 mm) was released, but that if late parr stages (larger than 70 mm) are released, enrichment is reported to have a positive effect on smolt migration survival. Here, we explored the effect of enrichment at two different stages of development. Both stages were reared and treated for 7 weeks (fry at 11–18 weeks and parr at 24–31 weeks after hatching) before tested for behavior. Responses known to be associated with exploratory behavior, activity, and stress coping were quantified by testing 18-week-old fry and 31-week-old parr in a six-chamber maze on 7 successive days after rearing in structurally enriched (plastic plants and tubes) or plain impoverished rearing environments. The data show that Atlantic salmon are sensitive to stimuli from structural enrichment when they are parr, but not when in the fry stage. Parr deprived of enrichment (control treatment) were reluctant to start exploring the maze, and when they did, they spent a longer time frozen than enriched parr, suggesting that deprivation of enrichment at this life can be stressful. Our data suggest that structural enrichment could have the potential to improve welfare for salmonids in captivity and for survival of released juvenile salmon if structural enrichment is provided at the parr stage and the fish reared for conservation are released at the parr stage.

## Introduction

Animals usually express behaviors that appear to be adapted to the environment in which they find themselves. In some cases, the development of adaptive behavior seems to be influenced and refined by early life experiences (1–3). For example, the developmental status of sensory systems such as vision depends on sensory experience in early life (4). This is particularly relevant for animals reared for release into the wild. Previous work with captive birds, mammals, and fish have already illustrated how increasing environmental complexity, often referred to as environmental enrichment, can increase behavioral and neuronal plasticity, improve cognitive performance, reduce stress responses, and increase survival in reintroduced species (3, 5–16). Some animals respond strongest to stimuli from environmental complexity at certain sensitive life stages and need specific stimuli for developing behaviors that are appropriate and effective in dealing with the changing conditions of life (2, 4, 17). Some species respond over a prolonged time period, while others are particularly sensitive for specific external stimuli over a short period at a specific life stage (4, 17). Others improve behavior after a few days of experience at any time during their first year of life (1).

Specific cues from external stimuli can shape certain behavioral phenotypes [e.g., (18–20)]. For example, rats showed enhanced cerebral plasticity after a few days of experience at any time during their first year of life, indicating that it is environmental stimuli *per se* that stimulated learning (1) and thus behavioral development rather than specific developmental phases. Others, such as chicks of ducks and hens, require auditory and visual stimuli within a few days after hatching to develop a social bond to their parents (21), indicating that for these species, auditory and visual stimuli are required at a specific life stage. The way external stimuli promote future decisions in animals seems thus to be context and taxa dependent.

A well-developed exploratory behavior may provide benefits during a life stage when animals shift into a new habitat or disperse over a larger area (22). If the mortality risk in the present habitat is increasing and/or prey abundance decreases, and an alternative habitat has better conditions, the theory of optimal habitat shift (23) predicts that it will be beneficial to shift habitat to minimize mortality risk per growth rate. For animals in which dispersal will reduce competition and provide access to novel resources, free-living dispersers should, according to the habitat selection theory, prefer new habitats that contain stimuli comparable to those experienced in their natal habitat (24–26). Some researchers suggest that stimuli of new habitats will have a stronger impact on preferences if encountered during a sensitive life stage (17, 24, 27–30). Others regard the phenomenon as more general and that experience with stimuli in an individual's natal habitat increases the probability that the individual later will select a habitat that contains similar stimuli (25, 31).

In Atlantic salmon (*Salmo salar* L.), eggs hatch in the nests in the spring and alevins remain there until they emerge as fry and settle in the vicinity of the nest site (32). In this vulnerable early life stage, a young fry increases its probability of escaping predation by aggregating and having a synchronous emergence pattern (29, 33). The young fry are drift feeders, but the propensity to actively search for prey increases with size (32) and involves expanding the spatial habitat to meet increased energy demands and changed feeding habits as the juveniles grow (34). In *S. salar*, a typical change is observed when juveniles develop from fry to parr: individuals longer than 70 mm (parr) have a larger spatial habitat than those shorter than 70 mm (fry). Foldvik et al. (22) showed that as the individuals grew, dispersal initially increases slowly until the fry reached 70–80 mm length (parr size), a size when dispersal rate almost doubled. It is known that juvenile Atlantic salmon shift feeding habit when they reach this size: Typically, as fry change from feeding primarily on benthic drift prey to become parr, they take up feeding stations and attack drifting invertebrates at the surface waters more often (30, 35, 36).

Here, we experimentally studied how the development of behaviors shaped by stimuli from structural enrichment depend on stage of exposure. We used juvenile Atlantic salmon (*S. salar*) in their first autumn. We reared for 7 weeks fry (11–18 weeks after hatching) and parr (24–31 weeks after hatching) in structurally enriched or plain impoverished (control) environments. After 7 weeks of treatment, we used six-chambered mazes and tested individual fish on 7 successive days for behaviors known to be associated with the propensity of exploratory behavior, with activity, and with stress coping. Exploratory behavior in fish is known to be stimulated by enrichment [e.g., (13, 14, 37, 38)] and to reduce stress response (15, 16). If stimuli from enrichment is important any time early in life for salmon, we predict that enrichment will stimulate exploratory behavior and stress coping in both fry and parr stages. We may however not rule out that Atlantic salmon could be a species with a sensitive life stage that need specific stimuli for proper behavioral developments to deal with the changing conditions of life as shown for animals from other taxa [e.g., (2, 4, 17)]. If so, an early life stage when dispersion is pronounced such as the parr stage (22) may need more sensitive experience than the fry stage, and the alternative hypothesis would then be that stimuli from structural enrichment are more relevant during a dispersal period (parr) than during a less mobile (fry) life stage.

## Materials and Methods

### Experimental Fish and Treatments

We follow the ARRIVE guidelines by Percie du Sert et al. (39) in our description of our experiments.

Salmon (*S. salar*) fry of wild origin (hatching date, April 20, 2015) were transferred from Voss hatchery to the experimental facilities at the University of Bergen (UiB), Norway, on two occasions. The first group arrived as fry on July 1, 2015, 11 weeks after hatching (*n* = 447, mean weight 0.5 g). The second group arrived when at the parr stage on September 30, 2015, 24 weeks after hatching (*n* = 501, mean weight 8.0 g). In both cases, the fish were randomly distributed into six identical treatment tanks (100 × 100 cm, water level 60 cm) by transferring five fish at a time into each of the tanks (in the end, three fish at a time) to minimize potential unequal distribution of fish caught early and late among the rearing tanks. The tanks were next numbered using tank number on paper labels (1, 2, 3, 4, 5, and 6) and treatment (three labels enriched and three labels control), and then sampling without replacement to match three tanks to be structurally enriched and three tanks to be plain impoverished control tanks. The structural enrichment consisted of plastic plants and structures made by plastic pipes fitted together using aquaria silicone. When the tanks were cleaned once a week, the structures were moved around. The same amount of time of disturbance was given to the enriched and control tanks. Physical conditions were identical in control and enriched tanks; 12:12 light/dark cycle was used with natural day light. The water had a temperature of approximately 12°C, the tanks had flow-through, and the water renewal was 4 L min^−1^. All tanks had the same flow direction, which was counterclockwise. Fish were fed continuously with commercial fish feed (EWOS microstart 40020 and microstart5), *ad libitum* in the light period, using automatic feeders (Hølland technology). All fish were kept under these conditions and reared for 7 weeks in enriched or control environments before we started the behavioral experiments. Fry were reared under these conditions 11–18 weeks after hatching and parr were reared 24–31 weeks after hatching.

We used a handheld dip-net and selected 18 enriched and 18 control fish from the rearing tanks: 6 from each of the three enriched rearing tanks and 6 from each of the three control rearing tanks and all of intermediate size. The mean length ± SE of fry was 5.0 ± 0.2 cm, and the mean body mass was 1.7 ± 0.1 g, whereas the mean length ± SE of parr was 10.3 ± 0.2 cm and the mean body mass was 16.0 ± 1.2 g. Four additional holding tanks were used to house these 36 fish: two were structurally enriched and two were plain (control). The 18 enriched fish were housed in one enriched tank before behavioral screening and in the other holding tank after screening. The 18 control fish were housed in one plain tank before screening and in the other plain tank after screening. In the holding tanks, the fish were hand-fed pellets two times per day, and bloodworms one time and in the evening.

Four days prior to the behavioral assays, the fish were individually marked to allow identification of test individuals. Each fish was first anesthetized using MS222 (80 mg 3L^−1^) and then marked with a unique combination of yellow and red fluorescent Visible Implant Elastomers (NMT INC Northwest Technology) on two to three dorsal and ventral positions.

### Experimental Arena and Experimental Test

**Figure 1:**
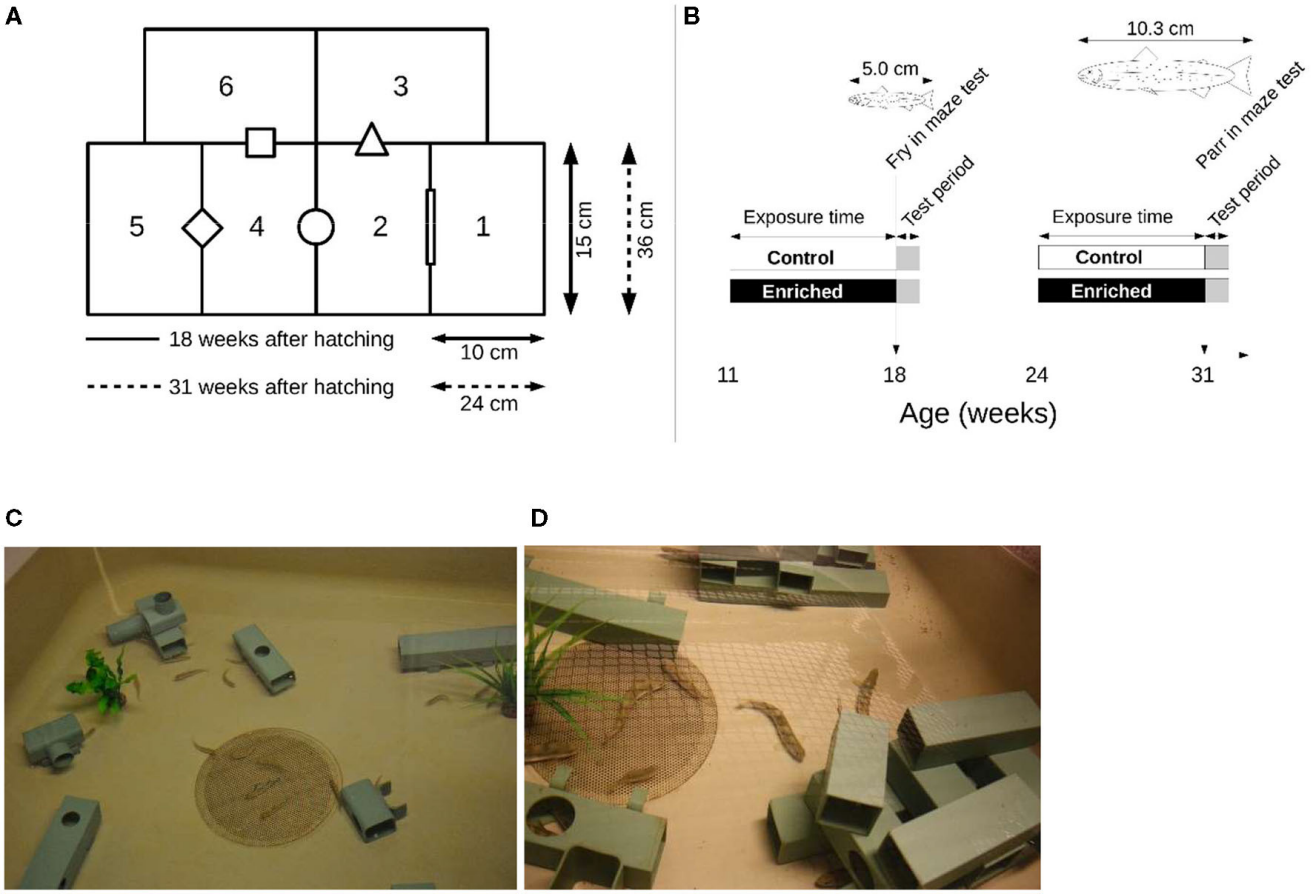
**(A)** Schematic representation of the mazes used to test exploratory behavior and activity of juvenile Atlantic salmon reared 11–18 weeks after hatching in structurally enriched or control environments and those reared 24–31 weeks after hatching. The ratio between the fish length and the chamber size was the same for both groups. **(B)** The timeline of the experimental setup. The water of the chambers was connected via openings that differed in geometrical shape. **(C)** Structures used when rearing fry, and **(D)** Structures when rearing parr for 7 weeks in enriched environments. Control fish were reared in identical tanks but without structures.

Four mazes were designed; two were used to test 18-week-old fry, and the two larger ones were used to test the 31-week-old parr. The ratios between fish length and possibility for horizontal movements in the maze were kept approximately the same. Both maze sizes consisted of six rectangular boxes (Figures 1A,B). The height of both mazes was approximately 11 cm. Chamber 1 of each maze was used as the start box, and its exit to chamber 2 was initially closed by an opaque removable door that was lifted remotely when each trial started. The water of the other five chambers was connected *via* openings that differed in geometrical shape.

Each maze was placed in the center of a larger holding tank (100 × 100 × 60 cm) and surrounded by water to keep temperature stable and to minimize injuries if the fish managed to escape its maze. The maze was covered with transparent glass to prevent fish from jumping out and to allow video recording from above. The water level in the maze was kept at approximately 11 cm. A video camera (LegraHFR560 Canon) was mounted above each maze.

Fish were familiarized to be moved from their holding tanks by allowing them to swim freely in all chambers in the maze in groups of nine for 1–2 h over 4 days prior to start of the behavior experiments. The fish had then access to food (red bloodworms *Chironomidae* fitted into a green ring using Vaseline fitted with metal piece to keep it on the bottom) in all chambers during acclimation, and there were no doors blocking any entrance. An air stone in one chamber provided aeration of the water. Unfortunately, four enriched and three control parr fish jumped out of their holding tank at night between two acclimation days and were removed from the experiments. Two enriched and two control fry jumped out of their holding tank at night between two test days, as also did three enriched and one control parr. The number of individuals tested all 7 days were therefore reduced to 16 enriched and control fry and 11 from enriched and 14 from control treatments for large parr.

At the start of each trial, an individual fish was collected in a handheld dip-net, and its identity was recorded using UV-light (VI-light 405 nm, 82 mV) on the Visible Implant Elastomers, and then it was carefully released into the start box. To keep handling stress to a minimum, we tested the fish in the order in which they were netted. Thus, the order of testing was different for each trial. A Canon LegraHFR560 camera, mounted 1.5 m above the center of the maze, recorded the trials. Fish could not see the observer, but the observer could view the fish and maze from the display on the camera. After 5 min acclimation, the start box door was opened remotely using a pulley. The test lasted 10 min. The fish had access to food in chamber six during the experiment. We tested the fish once per day over seven consecutive days to test if enriched and control fry and parr differed in the way they responded to the behavioral tasks. Seven days were chosen as previous experiments using salmon in the smolt stage showed different learning over this length of time (14). The water was replaced between each test fish. We used J-watcher and analyzed the videos of individual behavior. The video coder was blind to the fish treatment. Each fish had an ID consisting of three letters. These three letters were visible on a label on the video, but the video coder was not informed which codes belonged to which treatment groups. The following data were collected: (i) the time the test fish took to leave the start box, which, for fish, is a common proxy for exploratory behavior [e.g., (11, 40–42)]; (ii) the number of chamber changes; and (iii) the time the fish stayed still (froze) after it had left the start chamber.

### Ethical Note

All procedures have been completed according to the Norwegian Food safety Authority in compliance with “The regulation on the Use of Animals in Research with FOTS ID 7931.” After the trial on the last day, the fish were euthanized by an overdose of buffered MS222 (0.5 g L^−1^).

### Data Analysis

All statistics were performed using R v4.0.3 [(43), http://www.r-project.org].

For individual fish, we first calculated the cumulative time to leave the start chamber, cumulative number of chamber changes, and cumulative time spent frozen for seven successive experimental days. Cumulative time to leave the start chamber was used as a proxy for exploratory behavior, cumulative number of chamber changes was used as a proxy for activity, and cumulative time spent frozen was used as a proxy for fear. Cumulative data were chosen for the analysis since both consistency in behavioral differences between the treatment groups and the link between behavior and survival is best considered if accumulated over several observation times. For the continuous response variables “body mass,” “length,” “time to leave the start box,” and “time spent frozen,” we fitted linear mixed-effects models using the lme function from the nlme library of R (44). For analyses concerning the discrete response variable “number of chamber changes,” we fitted generalized linear mixed-effects models with Poisson error term using the glmmPQL function from the MASS library of R (45). Due to differences in both fish and maze size between the two age classes, we did separate analyses for the fry and parr life stages. In all models, “tank” was set as a random effect factor to account for the dependency structure caused by multiple fish in each of the six treatment tanks. “Treatment” (reared in structurally enriched or control environments) was specified as the predictor. We wanted to explore if motivation to explore and activity levels depended on stress coping in fry and parr. Therefore, we used the proxy for stress coping (time spent frozen), treatment, and interaction between these two as predictors in some additional analyses. All tests were done using cumulative data over 7 days.

## Results

Immediately after an individual had been transferred to the start chamber of the maze with a dip-net, all fish exhibited some level of fearfulness. Initially, the test fish froze before swimming a little around. Once the chamber was opened, the fish tended to move to the opening, standing still a little looking at the space outside the start chamber before entering the maze and swimming slowly around. Typical behavior in the maze was to change between swimming slowly around and standing still (freeze) while visiting different compartments of the maze.

There was no difference in size between structurally enriched and control fish when they were tested for behavioral differences. Fry did not differ in length [lme; *F*_(1, 4)_ = 0.71; *p* = 0.45; mean length 5.0 cm] or in body mass [lme; *F*_(1, 4)_ = 0.005; *p* = 0.96; mean body mass 1.7 g], and parr did not differ in length [*F*_(1, 4)_ = 0.11; *p* = 0.76; mean length 10.3 cm] or body mass [*F*_(1, 4)_ = 0.29; *p* = 0.62; mean body mass 16 g, respectively].

**Figure 2:**
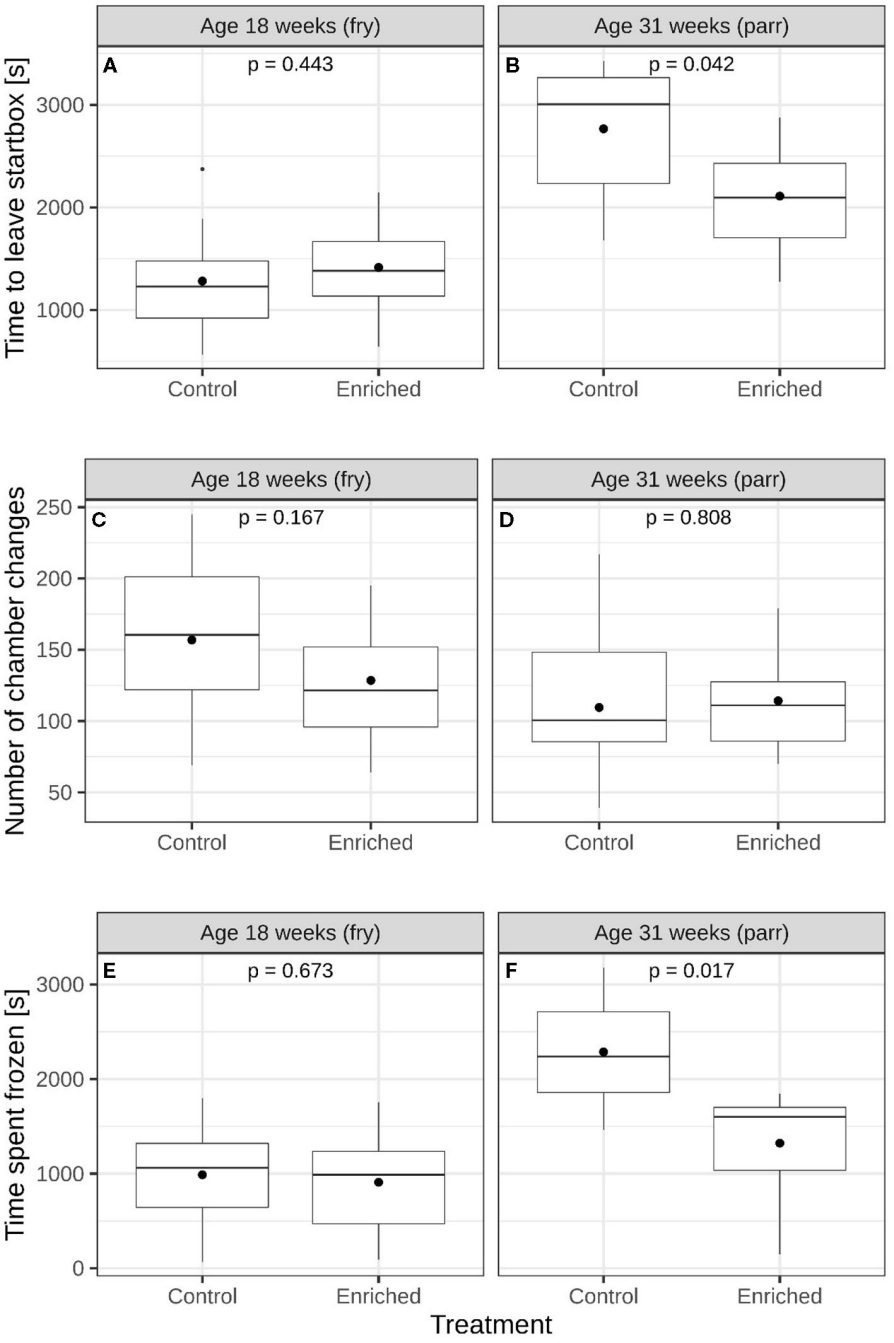
Comparisons of enriched reared and control reared juvenile Atlantic salmon (*Salmo salar*). Time to leave start box of **(A)** 18-week-old and **(B)** 31-week-old juveniles. Number of chamber changes of **(C)** 18-week-old and **(D)** 31-week-old juveniles. Time spent freezing of **(E)** 18-week-old and **(F)** 31-week-old juvenile salmon. The boxes show the medians and quartiles while the whiskers show the extremes within 1.5 times the interquartile range. Black circles represent mean values. The *p*-values in each panel represent a comparison of mean values between the two groups.

At 18 weeks of age, enriched and control fry did not differ in time to leave the start box [Figure 2A and Supplementary Figure 1A, lme; *F*_(1, 4)_ = 0.725, *p* = 0.443], the number of chamber changes (Figure 2C and Supplementary Figure 1C, glmmPQL; *t* = 1.685, *df* = 4, *p* = 0.167), or the time spent frozen [Figure 2E and Supplementary Figure 1E, lme; *F*_(1, 4)_ = 0.207, *p* = 0.673, respectively]. At 31 weeks of age, the enriched parr individuals left earlier [Figure 2B and Supplementary Figure 1B, lme; *F*_(1, 4)_ = 8.744, *p* = 0.042] and spent less time frozen [Figure 2F and Supplementary Figure 1F, lme; *F*_(1, 4)_ = 15.721, *p* = 0.017] in the maze test than control parr did. Enriched and control treatment groups did not differ with respect to the number of chamber changes (Figure 2D and Supplementary Figure 1D, glmmPQL; *t* = 0.260, *df* = 4, *p* = 0.808).

**Figure 3:**
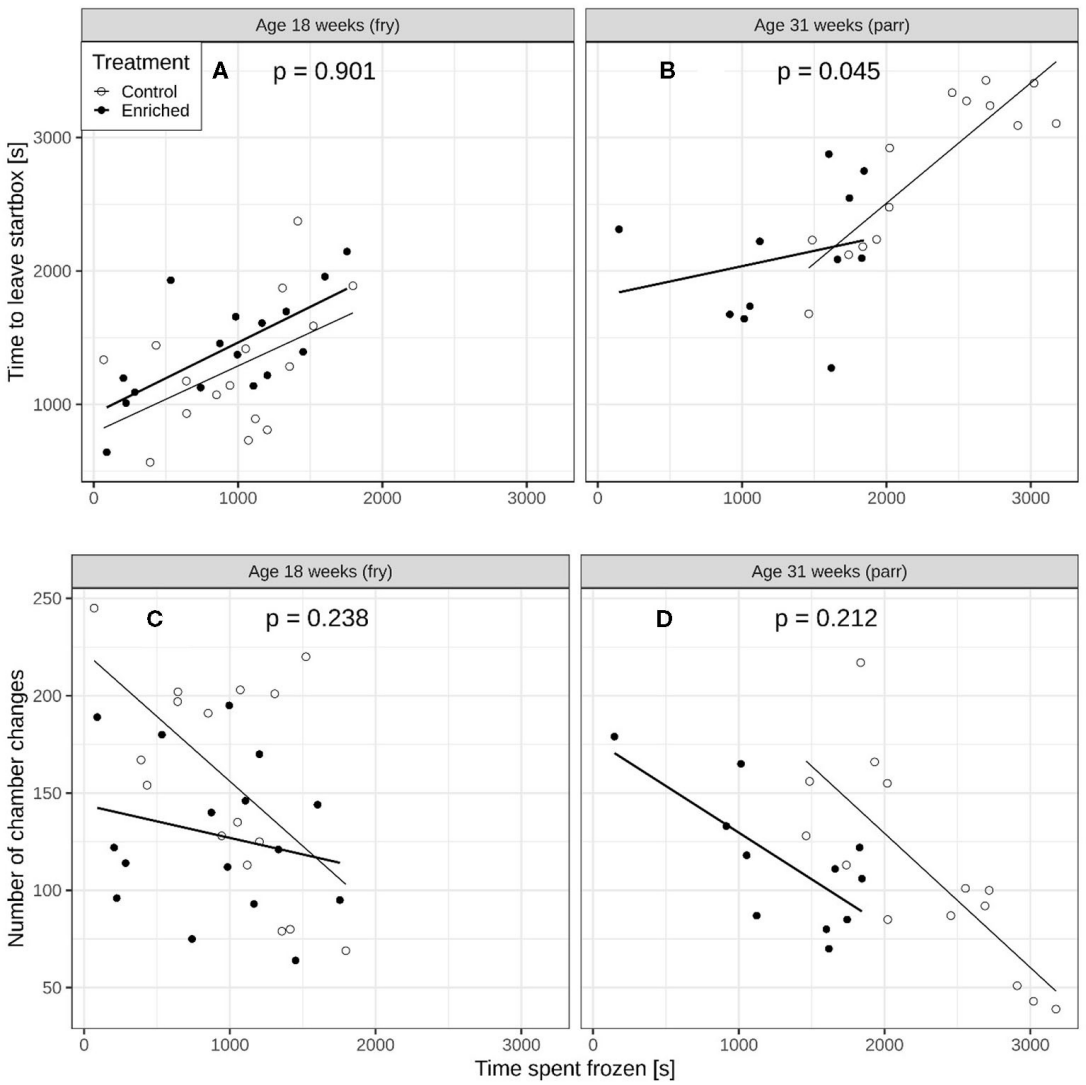
Comparisons of enriched reared and control reared juvenile Atlantic salmon (*Salmo salar*). Time to leave start box vs. time spent immobile for **(A)** 18-week-old and **(B)** 31-week-old juveniles. The *p*-values on each panel refer to the interaction term and represent a comparison of slope between the two regression lines. Number of chamber changes versus time spent immobile for **(C)** 18-week old and **(D)** 31-week old juveniles.

At 18 weeks of age, the effect of time spent frozen on the time to leave the start box [Figure 3A, interaction term from lme: *F*_(1, 24)_ = 0.016, *p* = 0.901] and on the number of chamber changes was similar for enriched and control fry (Figure 3C, interaction term from glmmPQL; *t* = 1.211, *df* = 24, *p* = 0.238). At 31 weeks of age, however, the effect of time spent frozen after they left the start box on time to leave was significantly weaker for the enriched parr compared to the control parr [Figure 3B, interaction term from lme: *F*_(1, 17)_ = 4.699, *p* = 0.045]. The effect of time spent frozen on number of chamber changes was, however, similar for the enriched and control parr (Figure 3D, interaction term from glmmPQL: *t* = 1.30, *df* = 17, *p* = 0.212).

## Discussion

These results reveal an interaction between experience from different types of environmental heterogeneity, age of experience, and subsequent behavioral responses. These results are in line with enrichment experiments on higher vertebrates [e.g., (2, 10, 19, 46)] and other fish species (11, 12, 47, 48) and also in line with experiments on later life stages of Atlantic salmon (14, 49, 50). In addition, these results follow the ideas of Immelmann (24) and Knudsen (17), suggesting that in animals having sensitive developmental periods, stimuli will have a stronger impact on the development of a behavioral response if it is experienced during a specific period/life stage. Our data show that 18-week-old enriched and control fry did not differ in exploratory behavior, activity, and stress coping, as indicated by the proxies used to study these behaviors (time to leave the start box, the number of chamber changes, and the time spent frozen). This suggests that stimuli from structural enrichment may not be relevant at the fry life stage. In parr, however, which were from the same cohort but both treated and tested when 13 weeks older (3 months later), the enriched individuals could be regarded as more exploratory as they left the start box earlier than control parr. Enriched parr did also show elevated stress coping compared to control fish as they spent less time frozen in the maze after they had left the start chamber than control parr did. Enriched and control parr had, however, similar activity as the number of chamber changes they did during the test did not differ.

Our data suggest that in Atlantic salmon, the parr seem to be more behaviorally plastic than the fry. Hence, stimuli for suitable exploratory behavior and stress coping would be needed at the life stage when Atlantic salmon juveniles typically expand their habitat and alter feeding habits. Although for all the parr, the longer they spent frozen once the door of the chamber was opened, the longer they took to leave the chamber, the enriched parr spent less time frozen than parr reared in impoverished control environments. As most fish exhibited some level of fearfulness just after transfer to the start box of the maze and where they initially froze before leaving, this could be interpreted as the start box was somewhat stressful for both treatment groups and similar for enriched and control fry. However, the control parr that were significantly more reluctant to leave the start box and also froze for a longer time after they had entered the maze than enriched reared parr (Figure 3B) appears to have been more fearful than enriched parr. Hence, a potential motivation of enriched reared parr for leaving the start box could be to leave a space associated with fear and seek for potential shelters. Presence of shelter has been shown to reduce stress in salmon and to reduce metabolic costs (15, 16, 51).

Previous researchers have shown that during the transition between fry and parr, dispersal rate increases and the feeding habits change from relying on benthic drift prey to more actively feeding on pelagic prey shallower in the water column (22, 30, 34–36, 52). Our data show that prior experience of stimuli from structural enrichment had a positive effect on our proxy for explorative behavior as enriched reared parr left the start box earlier than control parr that had been deprived from such stimuli. Previous studies have shown that enrichment causes decreased stress hormone levels in juvenile Atlantic salmon and Pacific salmon, although those studies did not investigate exploratory behavior *per se* (15, 16). It might be that the behavioral response in control parr could reflect higher stress level compared to those from structural enriched tanks, but further studies would be needed to investigate this in fuller detail. Our study was a pure behavior study and did not include physiological measures such as hormone levels in the test fish, though the behavioral responses we measured are known to be associated with exploratory behavior, activity, and stress coping.

Why did enriched reared salmon juveniles respond differently to stimuli from structural enrichment as fry and parr? Wild fry do experience complex habitats after emergence, but early in the fry life stage, wild fry of Atlantic salmon tend to stay fairly stationary in hiding places among the stones on the bottom, where they are supplied with small benthic crustacean prey transported *via* currents and eddies (32). The fry are small and have low energetic storage. For fry, prey capture and dispersing into a larger habitat would be too energetically costly and probably exceeding the potential benefit of encountering additional prey passing by. However, as fry grow, they build energy storage and become sufficiently large to have the capacity for successful capture of larger prey and also for dispersing further away from where they hatched [e.g., (53)]. As animals disperse, they will need to quickly and reliably be able to recognize a suitable habitat for settlement (25), using cues that resemble those experienced earlier (26).

Not all studies have reported positive effects of structural enrichments. Whether structural enrichment promote exploratory behavior seems to depend on the context and the model species used for the study. For example, structurally enriched reared cod (*Gadus morhua*) juveniles explored more than control juveniles reared in impoverished tanks (11), while enriched and control reared juvenile steelhead salmon (*Oncorhynchus mykiss*) did not differ in their exploratory behavior (54). *O. mykiss* was also studied by Bergendahl et al. (38) who found that both the timing and duration of experience from structural enrichment influenced the strength of the behavioral response, but enrichment had no effect on anxiety-related behaviors for this species.

Our overall findings are relevant for conservation biology and the welfare of fish. The use of enrichment is considered important in captivity for all stages of animal's life. Fishes are important laboratory animals and many species are housed in captivity as model organisms for research (55), and others are kept in captivity for aquaculture and ornamental pets. Housing conditions are important in the welfare of captive animals, of which environmental enrichment is an important component (48). Previous studies have demonstrated that environmental and social enrichment promote behavior and cognition in animals in general. Releases of hatchery-reared salmonids deprived from such stimuli into natural environments tend to be unsuccessful. Therefore, enriched rearing has, for the last decades, been widely used as a tool for managing stress in captive fish and with the ambition of producing fish with better survival in the wild. Studies that have released enriched reared early stages of salmonids (i.e., younger than 18 weeks; the fry life stage) did not find higher survival after release (56–58). Solås et al. (59) who released 12- to 17-week-old fry in three different years report higher survival from predation mortality 48 h after release only in the year when 17-week-old fry were released, but enriched fish did not have higher survival 12 weeks after release of fry. A few release experiments using older Atlantic salmon parr at release have, however, demonstrated higher smolt migration survival of enriched reared compared to 11-month-old (50) and 24-month-old (49) control fish. Previous findings and our data suggest that structural enrichment could have the potential to improve welfare for salmonids in captivity and for survival of released juvenile salmon at later stages than the fry stage. Further studies will be required to investigate the timing, amount, and type of environmental enrichment that produce the best welfare for captive fish and survival of released ones. At present, there is a lack of knowledge on what enrichment to provide for most fishes housed in captivity or the effectiveness of different kinds of enrichment that is used, and there is a need for the development of standardized enriched housing that can provide welfare benefits for captive fish at all life stages (48).

## Data Availability Statement

The raw data supporting the conclusions of this article will be made available by the authors, without undue reservation.

## Ethics Statement

The animal study was reviewed and approved by Norwegian Food safety Authority in compliance with the regulation on the Use of Animals in Research with FOTS ID 7931.

## Author Contributions

IA and ASa designed the study. IA reared the fish and did the experiments under supervision from ASa. IA did the initial analyses under supervision from KJ. KJ did the final statistical analyses and made all figures. IA wrote the first draft of the manuscript with input from ASa, KJ, and ASk. ASa revised the manuscript with input from IA, KJ, and ASk. All authors have approved the final version.

## Conflict of Interest

The authors declare that the research was conducted in the absence of any commercial or financial relationships that could be construed as a potential conflict of interest.

## Publisher's Note

All claims expressed in this article are solely those of the authors and do not necessarily represent those of their affiliated organizations, or those of the publisher, the editors and the reviewers. Any product that may be evaluated in this article, or claim that may be made by its manufacturer, is not guaranteed or endorsed by the publisher.

## Supplementary Material

Supplementary Figure 1 Comparisons of control and enriched reared juvenile Atlantic salmon (*Salmo salar*). The lines show cumulative values at the individual level over seven days of testing, where grey and black lines represent control and enriched reared fish, respectively. Time to leave start box of **(a)** 18-week old fry and **(b)** 31-week old parr. Number of chamber changes of **(c)** 18-week old fry and **(d)** 31-week old parr. Time spent freezing of **(e)** 18-week old fry and **(f)** 31-week old parr. Open and solid circles represent mean values for control and enriched reared fish, respectively.
