# Supplementary material for: Ontogenetic Change in Behavioral Responses to Structural Enrichment From Fry to Parr in Juvenile Atlantic Salmon (Salmo salar L.)
Source: Front Vet Sci. 2021 Jul 26;8:638888. doi: 10.3389/fvets.2021.638888 (PMC8350771; doi:10.3389/fvets.2021.638888)
Supplement: Supplementary Figure 1 — Comparisons of control and enriched reared juvenile Atlantic salmon (Salmo salar). The lines show cumulative values at the individual level over seven days of testing, where grey and black lines represent control and enriched reared fish, respectively. Time to leave start box of (a) 18-week old fry and (b) 31-week old parr. Number of chamber changes of (c) 18-week old fry and (d) 31-week old parr. Time spent freezing of (e) 18-week old fry and (f) 31-week old parr. Open and solid circles represent mean values for control and enriched reared fish, respectively. [file Presentation_1.pdf]

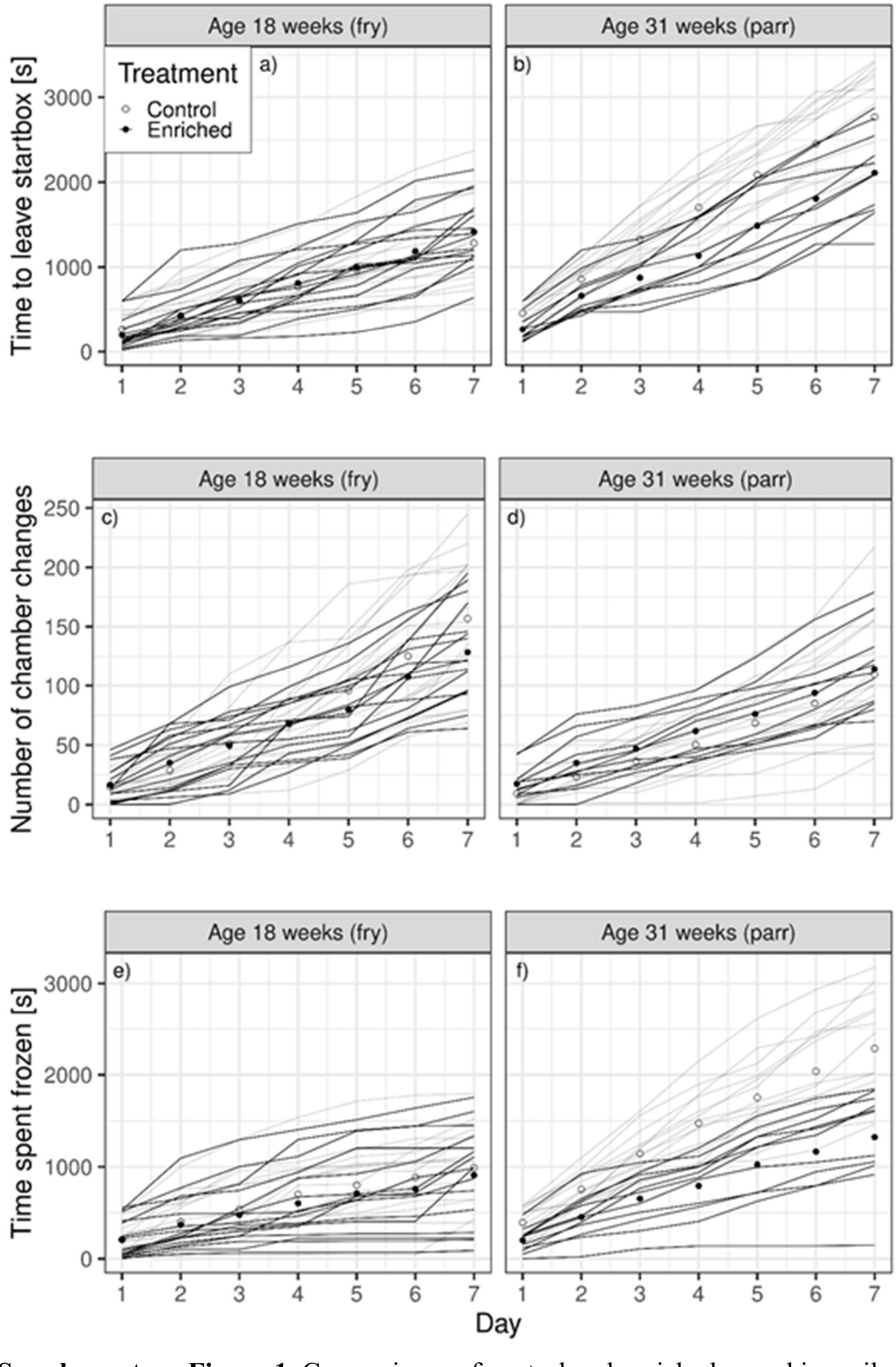

Supplementary Figure 1. Comparisons of control and enriched reared juvenile Atlantic salmon (*Salmo salar*). The lines show cumulative values at the individual level over seven days of testing, where grey and black lines represent control and enriched reared fish, respectively. Time to leave start box of (a) 18-week-old fry and (b) 31-week-old parr. Number of chamber changes of (c) 18-week-old fry and (d) 31-week-old parr. Time spent freezing of (e) 18-week-old fry and (f) 31-week-old parr. Open and solid circles represent mean values for control and enriched reared fish, respectively.
