# Supplementary material for: Ontogenetic Change in Behavioral Responses to Structural Enrichment From Fry to Parr in Juvenile Atlantic Salmon (Salmo salar L.)
Source: Front Vet Sci. 2021 Jul 26;8:638888. doi: 10.3389/fvets.2021.638888 (PMC8350771; doi:10.3389/fvets.2021.638888)
Supplement: Supplementary file 2 [file Data_Sheet_1.PDF]

The behavior in many species of animals is often influenced by what they experience early in life. One such factor, which is known to affect behaviour, is the complexity of the environment, often called structural enrichment. We studied the fry and parr life stages of Atlantic salmon reared in structurally enriched or plain impoverished nursery environments. Our data suggest that behaviour of the parr life stage is sensitive to environmental complexity, while the fry stage is not. Parr deprived of enrichment were less bold and showed a lower degree of exploratory behavour as compared with parr that had been kept in a more complex environment. Among the fry we found no such differences. We discuss these results in view of the timing and relevance of external stimuli for captive housing of fish, for fish survival after released for conservation, and for fish welfare.
